# Beyond adhesion: emerging roles for integrins in control of the tumor microenvironment

**DOI:** 10.12688/f1000research.11877.1

**Published:** 2017-08-31

**Authors:** Whitney Longmate, C Michael DiPersio

**Affiliations:** 1 Department of Regenerative and Cancer Cell Biology, Albany Medical College, Albany, New York, USA; 2 Department of Surgery, Albany Medical College, Albany , New York, USA

**Keywords:** Integrin, Cancer Therapy, Cell Signalling

## Abstract

While integrins were originally discovered as cell adhesion receptors, recent studies have reinforced the concept that integrins have central roles in cancer that extend far beyond controlling cell adhesion and migration. Indeed, as transmembrane cell surface receptors that occupy a critical position at the interface of cellular and extracellular interactions and are capable of both “inside-out” and “outside-in” signaling, integrins are uniquely poised to regulate the cell’s ability to promote, sense, and react to changes in the tumor microenvironment. Moreover, integrins are present on all cell types in the tumor microenvironment, and they have important roles in regulating intercellular communication. Decades of promising pre-clinical studies have implicated certain integrins as attractive therapeutic targets in the cancer clinic. Nevertheless, results of the few clinical trials that target integrins in cancer have thus far been disappointing. Importantly, these clinical failures likely reflect the emerging complexity of individual and combinatorial integrin function within both tumor cells and other cell types of the tumor microenvironment, together with a need to explore integrin-targeting agents not just as monotherapies but also as adjuvants to more conventional radiotherapies or chemotherapies. In this review, we will examine recent advances toward understanding how integrins regulate cancer progression, including their roles in intercellular communication and modulation of the tumor microenvironment. Additionally, we will discuss factors that underlie the limited efficacy of current efforts to target integrins in the cancer clinic as well as potential strategies to overcome these challenges.

## The integrin family of cell adhesion receptors

Integrins are the major receptors on the cell surface for adhesion to the extracellular matrix (ECM) ^1^. All members of the integrin family are heterodimeric, transmembrane glycoproteins that consist of an α and a β subunit, each with a cytoplasmic domain, a single-pass transmembrane domain, and a large extracellular domain. The dimerization of 18 α subunits and 8 β subunits in limited combinations leads to the formation of 24 different integrin pairs with distinct and often overlapping ligand-binding specificities. As a group, integrins interact with a plethora of ECM proteins, to which they bind via their extracellular domains ^1^. Simultaneously, integrins interact with cytoskeletal proteins via their cytoplasmic domains to mediate a physical, transmembrane linkage of the ECM to the cytoskeleton, which is critical for controlling cell shape, polarization, and motility ^1– 5^.

It has long been known that integrins can also interact directly or indirectly with a wide variety of signaling effectors, thereby functioning as conduits of bidirectional signal transduction across the cell membrane ^1, 2, 6, 7^. Indeed, integrins can regulate intracellular pathways in response to ECM binding or other extracellular cues (i.e. “outside-in” signaling) while cytoplasmic interactions can regulate the activation state of an integrin to alter its affinity for extracellular ligands (i.e. “inside-out” signaling) ^1, 6, 8^. Such integrin-mediated signal transduction controls a wide variety of cell functions that are important for both normal and pathological processes, including survival, proliferation, migration, ECM remodeling, and gene expression ^1, 3, 6^.

Integrins play critical roles in cancer progression, where they are expressed on the surfaces of both tumor cells and all other cell types present in the tumor microenvironment (TME). From within these distinct cellular compartments, integrins regulate functions of both tumor cells and stromal cells that promote tumor growth and malignant progression, including the above-mentioned cell-autonomous functions, as well as communication between different cell types of the TME (for reviews, see 9– 14). Thus, as bidirectional signaling receptors that regulate both cell-mediated changes to the microenvironment and cellular responses to those changes, integrins are attractive targets for therapeutic strategies against cancer. Indeed, the general concept of targeting integrin function to treat cancer or other diseases is already well established ^10, 11, 15, 16^. Here, we will briefly examine the current state of integrin-targeting cancer therapeutics, as well as limitations and knowledge gaps in the field that must be overcome to better exploit integrins as efficacious cancer targets. In particular, we will consider recent advances in understanding the complex roles that integrins play in modulating the TME and how this knowledge may impact the development of strategies to target integrins in the clinic, either as monotherapies or as adjuvants to conventional radiotherapies or chemotherapies.

## Pro-tumorigenic integrin signaling in cancer cells

Generally, integrin-mediated signal transduction occurs through direct or indirect interaction of the integrin cytoplasmic domain with cytoplasmic signaling effectors, which in some cases may be aided through lateral interactions with other integrin-associated cell surface proteins such as tetraspanins, caveolin, or urokinase-type plasminogen activator receptor (uPAR) ^11, 17– 19^. It has long been known that various intracellular signaling effectors can propagate outside-in integrin signaling, as reviewed in detail elsewhere ^6^. Among the best-studied integrin-mediated signaling effectors in cancer cells are the non-receptor tyrosine kinases focal adhesion kinase (FAK) and Src. FAK and Src are both elevated in solid tumors of many origins, including breast, epidermal, and colon cancers, where they contribute to tumor growth and malignancy ^20, 21^. Following integrin-mediated cell adhesion, FAK is activated by an autophosphorylation event on Y397, which generates a high-affinity binding site for the SH2 domain of Src. Once bound, Src mediates the phosphorylation of additional tyrosine residues of FAK, creating binding sites for other signaling and adaptor molecules. The activated FAK/Src scaffold thereby links integrins to downstream signaling effectors such as the Rac1 GTPase or the mitogen-activated protein kinases (MAPKs, i.e. ERK1/2, JNK, or p38).

Such signaling pathway activation can augment tumor cell migration, proliferation, survival, and gene expression, thus contributing to tumor growth and metastasis and implicating these pathways as potential therapeutic targets ^22, 23^. Interestingly, results of studies in breast and squamous cell carcinoma models suggest that integrins, possibly through FAK signaling pathways, promote tumorigenesis by maintaining the pool of slow-cycling stem cells that are the primary targets of oncogenic transformation ^24, 25^. Importantly, integrin signaling (in some cases through FAK) has been shown to mediate resistance to conventional therapies such as radiation and chemotherapy ^26– 30^, indicating that the inhibition of integrins may prove to be most beneficial in the adjuvant setting to render other therapies more effective (as reviewed in 30). On the other hand, the stimulation of integrin signaling using agonists has also been shown to improve the response of melanoma to chemotherapy, suggesting that in some cancers the activation, rather than inhibition, of certain integrins may be beneficial, as reviewed elsewhere ^31^.

## Roles for integrins in the regulation of the tumor microenvironment

It is now well accepted that transformed cells, in and of themselves, are not sufficient to generate appreciable tumors with metastatic potential. Rather, cancer development additionally requires a permissive TME, and recent work in the field of cancer biology has begun to focus more on non-tumor cell components of the stroma as drivers of malignant progression (for reviews on this topic, see 32– 34). As bidirectional signal transducers that are expressed on all cell types within the TME, integrins have emerged as regulators of tissue remodeling and intercellular communication between different cell types that drive tumor-supporting features of the tumor niche. In the following sections, we will focus on the roles that integrins play in modulating the TME through control over matrix remodeling and paracrine communication between distinct cell types. We will also discuss the importance of considering the complexity of these roles when attempting to circumvent the challenges associated with integrin-targeting therapies in the cancer clinic.

### Integrin regulation of tumor cell invasion and metastasis

The journey of a cancer cell from the primary tumor to a distant metastatic site is an arduous one. To survive this journey, cancer cells must be able to disassociate from the primary tumor, invade adjacent stroma, intravasate the vasculature (either blood or lymph), survive circulation, then extravasate from the vasculature into a hospitable secondary site. Each step of this metastatic process selects for those tumor cells that have acquired genetic and/or epigenetic traits that allow them to survive, grow, and invade in the face of a dynamic microenvironment and to degrade or remodel basement membranes/ECM along the way ^35^.

Integrins play vital roles in cancer metastasis during every leg of the metastatic cascade ^36, 37^. Indeed, integrins are critical in mediating adhesion dynamics and migration during metastasis through their ability to regulate both physical interactions with the ECM and actin cytoskeletal dynamics ^3– 5^. Certainly, roles for integrins extend well beyond cell adhesion and migration in their capacity to stimulate intracellular signaling pathways that promote proliferation and survival ^6, 38^. Consistently, the upregulation or persistence of certain integrins has been generally linked to tumor invasiveness ^9, 12^. For example, the expression on certain cancer cells of a number of integrin subunits, including α3, α5, α6, αv, β1, β3, and β4, has been linked to invasive and metastatic potential ^39– 42^.

With regard to the metastatic spread of cancer, the ability of integrins to control cell adhesion and migration alone is insufficient to drive invasion/metastasis, since an intact basement membrane and stromal ECM provides a physical barrier that precludes a normal cell’s ability to invade local and metastatic sites. Thus, a critically important function of integrins is their additional ability to regulate matrix organization and remodeling through both proteolytic and non-proteolytic mechanisms ^12, 43^. Indeed, their dual roles in modulating both cell migration and ECM remodeling makes them astute mediators of invasion and metastasis and further implicates them as viable targets for therapeutic intervention.

Numerous studies have shown that integrins can promote cell invasion through controlling the expression and/or proteolytic function of matrix metalloproteases (MMPs) and other extracellular proteases that directly degrade ECM proteins, as reviewed extensively elsewhere ^12, 44^. Additionally, it is important to keep in mind that integrin-mediated modulation of MMPs and other matrix-modifying enzymes can impact tumorigenesis through the release of ECM-bound reservoirs of stored growth factors into the TME ^45, 46^. In these ways, the impact of integrin-dependent protease expression/function can have profound effects on cancer progression, including by mediating intercellular communication between cells in the TME via proteolytic activation of growth factor signaling ^47– 49^.

### Integrin regulation of tumor angiogenesis

Since intratumoral vessel generation is essential for appreciable tumor growth, many anti-cancer therapeutics have focused on the inhibition of endothelial cell functions required for angiogenesis rather than directly targeting tumor cells themselves ^50, 51^. It is well documented that several endothelial integrins, such as certain αv integrins and α5β1, have complex roles in promoting tumor angiogenesis through the regulation of endothelial cell migration and proliferation ^52– 54^ and that simultaneous inhibition of both αvβ3 and α5β1 may be required for potent anti-angiogenic effects in some cases ^55^. Interestingly, another recent study showed that endothelial-specific deletion in mice of key RGD-binding integrins (i.e. α5β1 and αvβ3/β5) failed to reduce tumor angiogenesis, indicating compensatory involvement of other integrins and illustrating the potential importance of targeting multiple integrins at once to inhibit tumor angiogenesis ^56^. Indeed, pro-angiogenic roles for the collagen-binding integrins α1β1 and α2β1 have been described ^57^. Additionally, integrin α4β1 and its ligand, vascular cell adhesion molecule-1 (VCAM-1), have been demonstrated to be important in vessel formation in both tumor and non-tumor settings ^58, 59^. Other potentially important receptors on endothelial cells include the laminin-binding integrins α3β1, α6β1, and α6β4 ^60, 61^; however, their roles in angiogenesis are less well explored. Results of genetic studies indicate that while endothelial β4 promotes tumor angiogenesis ^62^, the α6 and α3 integrin subunits have suppressive roles in tumor angiogenesis ^63, 64^. Collectively, these findings may reflect opposite roles for α6β1 and α6β4 in tumor angiogenesis ^62, 63^, emphasizing the importance of delineating between integrin heterodimers that share a subunit. Clearly, elucidating the complex roles of endothelial integrins in the regulation of tumor angiogenesis will require further studies that take into consideration the complex interplay between the various integrins that are expressed by the vasculature.

Some integrins on tumor cells can promote angiogenesis in a paracrine fashion by inducing the secretion of soluble factors that stimulate endothelial cells, emphasizing the importance of targeting integrins within the appropriate cellular compartment of the tumor. For example, we and others have shown that integrin α3β1 in breast cancer cells can stimulate the expression of MMP-9 ^65^ and Cox-2 ^66^, indicating a role for this integrin in generating pro-angiogenic paracrine signals from tumor cells to endothelial cells. This function reflects a similar role for α3β1 in wound epidermis, where it regulates the expression of pro-angiogenic factors that induce the paracrine stimulation of angiogenesis ^67^. Similarly, integrin α6β4 has been shown to enhance the expression of vascular endothelial growth factor in breast carcinoma cells, impacting not only angiogenesis but also tumor cell survival ^68^. Paradoxically, as mentioned above, the expression of α6 or α3 on vascular endothelial cells has been associated with the repression of pathological angiogenesis ^63, 64^, illustrating the important point that roles for specific integrins are dependent on cellular context and that these roles are not necessarily the same across distinct cellular compartments. Additionally, our recent study demonstrated that integrin α9β1 in the epidermis can inhibit α3β1-dependent, paracrine stimulation of wound angiogenesis ^69^, indicating that cross-suppression/transdominant inhibition amongst integrins ^70– 72^ adds another level of complexity to their roles in regulating angiogenesis that must be considered when developing integrin-targeting strategies.

### Integrin regulation of intercellular communication within the TME

While it has been well established for some time that a pro-angiogenic microenvironment is critical for cancer progression, contributions from other stromal components of the TME had been underappreciated until more recently. For example, tumor-associated fibroblasts (TAFs)/cancer-associated fibroblasts (CAFs) and tumor-associated macrophages (TAMs) had been viewed largely as passive stromal constituents or bystanders to the ravaging effects of tumorigenesis and metastasis. On the contrary, critical roles for these tumor-associated stromal cells in cancer progression are now beginning to be understood ^32, 34, 73, 74^. In the preceding section, we discussed recent evidence that certain integrins (e.g. α3β1) can control paracrine communication from tumor/epithelial cells to endothelial cells of the vasculature. It seems likely that such roles for integrins in intercellular communication will extend to other tumor-associated stromal cells that contribute to a tumor-permissive microenvironment.

It is now well known that TAFs/CAFs promote invasion and metastasis in several cancer types, owing in large part to their myofibroblast-like traits that generate a stiff ECM ^75– 77^. There is evidence that some integrins can contribute to the generation of stiff ECM. For example, integrin α2β1 ligation by collagen has been shown to regulate lysyl oxidase expression in cardiac fibroblasts ^78^, raising the possibility that integrins on TAFs have similar roles in promoting collagen crosslinking and ECM stiffness. Furthermore, as mediators of outside-in signal transduction, integrins play important roles in mechanosensing in response to ECM stiffness ^79^. Indeed, matrix stiffening has been demonstrated to promote tumorigenesis, at least in part, through the activation of integrin signaling ^80^. This potential for integrins to both promote and respond to ECM stiffening is consistent with their central role in the “dynamic reciprocity” that has long been known to exist between cells and the ECM ^81^.

While ECM stiffness is certainly important in tumorigenesis ^82^, it has become apparent that the roles of TAFs extend beyond the promotion of a desmoplastic TME to include the generation of paracrine signals to other cell types. In some cases, the response of the target cell to signals generated by TAFs (or other mesenchymal cells of the stroma) is mediated by integrins. For example, it was recently demonstrated in a gastric cancer model that activation of CXCL12/CXCR4 signaling in TAFs promotes the clustering of β1 integrins on tumor cells, thereby activating FAK signaling and promoting invasion ^83^. Similarly, β1 integrins were essential in tamoxifen-resistant breast cancer cells for migration and epithelial–mesenchymal transition that was induced by signals from TAFs ^84^. In another example, CD151 (a tetraspanin that complexes with certain laminin-binding integrins) was recently found to mediate pro-tumorigenic responses of prostate cancer cells to signals from tumor-associated osteoblasts ^85^. Additionally, some tumor cell integrins may activate pro-invasive proteolytic cascades through interactions with stromal cells. For example, the interaction of integrin α6β1 expressed on pancreatic cancer cells with uPAR expressed on stromal fibroblasts was linked with activation in the latter cells of the uPAR–uPA–MMP-2 proteolytic cascade, thereby aiding in tumor progression ^86^.

TAMs have also been linked to tumor progression in particular through their ability to promote tumor angiogenesis and invasion, although the molecular underpinnings of this link have just begun to be elucidated ^87– 89^. Importantly, some recent studies have implicated integrins in regulating the ability of TAMs to promote tumor progression. For example, an osteopontin-rich matrix activates TAMs in a melanoma model through ligation of integrin α9β1, leading to prostaglandin E2 production by TAMs that then stimulates the migration of both endothelial and cancer cells ^90^. Similarly, the ECM protein periostin, secreted by glioblastoma stem cells, may promote TAM recruitment to tumors via activation of αvβ3 on the latter cells ^91^. Additionally, integrin β2 interactions have been implicated in TAM-driven dissemination of tumor cells in an *in vitro* microfluidic model of lung adenocarcinoma ^92^.

## Integrins as targets for anti-cancer therapy

Integrins have several characteristics that make them attractive anti-cancer targets. First, they are generally important for mediating numerous cellular functions in all stages of cancer progression, including tumor cell proliferation, survival, angiogenesis, invasion, and metastasis ^9, 10, 38^. Additionally, as cell surface receptors, the integrins are relatively accessible by targeting agents. Finally, the broad impact on cell function of integrin-mediated signaling indicates that targeting a single integrin, or group of integrins, might generate a combinatorial and potentially potent effect. Unfortunately, these same broad functions of integrins, which occur in both normal cells and tumor cells, also make them difficult to exploit as targets in the clinic. The remainder of this review will discuss integrin-targeting strategies and the associated challenges as we move forward in exploiting these targets more efficaciously in the cancer clinic, including their development as adjuvant targets in currently used therapies.

### Clinical limitations of integrin-targeting anti-cancer therapeutics

From around the late 1990s to date, pre-clinical studies performed in a wide variety of cancer models have demonstrated that blockade or suppression of certain integrins can inhibit tumor growth or metastasis ^9, 10, 38, 93^. Outside the cancer clinic, a number of integrin-based therapeutics have progressed to phase II or phase III clinical trials, and some have had significant benefits for patients suffering from a number of disorders, including multiple sclerosis, thrombotic complications, and inflammatory bowel disease (IBD) (for excellent reviews on this topic, see 16, 31, 94). As one example, vedolizumab, an antibody against integrin α4β7, is approved for use in the United States, Canada, and Europe for patients with either subtype of IBD (ulcerative colitis or Crohn’s disease), both of which are potential precursors to gastrointestinal malignancies ^94, 95^. With regard to cancer, the concept of targeting integrins in the clinic using small molecule integrin antagonists (SMIAs) was realized when the integrin-blocking RGD mimetic Cilengitide entered clinical trials after performing well pre-clinically ^16^. Unfortunately, the transition of Cilengitide to the cancer clinic as a monotherapy has been met with little success ^96, 97^, which may be attributable in large part to several limitations. First, RGD mimetics target only the subgroup of integrins that bind RGD-containing ligands (e.g. fibronectin, tenascin, and vitronectin) ^1^ while failing to block other potentially important integrins that bind non-RGD ligands such as laminins. Indeed, the laminin-binding integrins remain unexplored as clinical targets despite abundant pre-clinical evidence that supports their participation in tumor growth and metastasis ^11, 15, 24, 66, 68, 98– 103^. Additionally, as discussed more below, integrin-targeting agents may prove to be most effective in the cancer clinic as adjuvant therapies alongside more conventional chemotherapy or radiation treatments ^30, 104– 106^.

Importantly, while Cilengitide was conceptualized to target RGD-binding integrins on endothelial cells to inhibit tumor angiogenesis, perhaps through abrogation of the FAK/Src/AKT pathway ^10, 107^, this approach is complicated by the presence of these integrins on additional cell types within the TME. For example, a recent study in a glioblastoma multiforme model showed that treatment with an RGD peptide also inhibits αvβ3 on TAMs, restricting their recruitment to the TME ^91^. Accordingly, moving forward it will be important to consider the potential effects of integrin-targeting agents on various cells within the tumor milieu as a whole. This approach is especially important considering that integrin expression profiles show overlap between distinct cell types and that integrins play important roles in paracrine crosstalk between different cells in the TME (discussed above).

An additional challenge is the necessity to create more directed therapeutic strategies that target key integrins within the appropriate cellular compartment(s) of the tumor. For example, while several current strategies rest on the premise of targeting endothelial cells to suppress tumor angiogenesis, other strategies that target integrins on tumor cells, or other stromal cells, remain relatively underexplored from a clinical perspective despite strong pre-clinical evidence to support this approach. Additionally, while many studies of integrin function in cancer have provided strong support for focus on the β1 integrins as a group, more attention must be paid to the functional diversity among the 12 distinct αβ heterodimers that comprise the β1 integrin subfamily ^1^. Indeed, it has long been known that individual β1 integrins have different expression patterns in many tumors ^108^, and studies have demonstrated diverse or opposing roles for distinct integrins. For example, α2β1 is a suppressor of metastasis in breast cancer cells ^109^, while α3β1 and α6β1 are pro-tumorigenic or pro-metastatic in breast cancer ^66, 102, 110, 111^. Moreover, some integrins may have pro-tumorigenic roles in some cancers but anti-tumorigenic roles in other cancers, as has been shown for α3β1 in breast or skin versus prostate cancer, respectively ^24, 66, 112^, indicating that the cancer context matters in terms of predicting the effects of inhibiting a particular integrin. Indeed, there are many additional examples wherein the ability of specific β1 integrins to perpetuate hallmarks of malignant progression can vary between different types of cancer, as recently reviewed ^113^.

### Minding the microenvironment: the future of integrin-targeting therapies

Despite the successful use of different integrin-targeting agents in pre-clinical cancer models, efforts to translate these findings to the cancer clinic have so far failed. This discordance most likely reflects the increasingly appreciated complexity of the integrin family and the knowledge gaps in the field that must be overcome for effective exploitation of integrins as therapeutic targets. Moreover, as already mentioned, a number of studies have indicated that targeting certain integrins may sensitize cancer cells to radiotherapy or other chemotherapies ^104, 105, 114^, suggesting that integrin-targeting agents may be most effective in the cancer clinic when administered as adjuvants to supplement more traditional cancer therapeutics.

As discussed in the preceding sections, current literature clearly supports critical roles for integrins in cell-mediated modulation of the microenvironment that drives normal tissue remodeling or contributes to pathological tissue remodeling. It is also likely that individual integrins have distinct but collectively critical roles in controlling intercellular communication within the TME based on mounting evidence described above that integrins can regulate paracrine signals that emanate from the epithelium or stroma in normal and pathological tissue states. Therefore, identification of the full spectrum of integrins that is expressed on cells within the TME, as well as improved understanding of the combined roles that different integrins play from within these distinct cell types, will be critical for effective exploitation of integrins as anti-cancer targets.

In addition to SMIAs discussed above, there are other integrin-targeting strategies that might eventually be exploited in the cancer clinic. An example is the use of aptamers, which act as RNA ligands that can be used to antagonize the protein targets to which they are directed (for a review on the use of aptamers in targeted therapies, see 115). Interestingly, a recent study demonstrated that an aptamer directed at integrin α6β4 caused reduced adhesion of PC3 prostate cancer cells to laminin-332 ^116^. Internalization of this aptamer was also demonstrated, suggesting potential utility (i.e. beyond adhesion-blocking) for the delivery of siRNA, microRNA, or toxins to α6β4-decorated cancer cells ^116^. Furthermore, an aptamer that targets an epidermal growth factor receptor (EGFR)–integrin αvβ3 complex was shown to impair αvβ3-dependent adhesion, as well as the matrix-induced interaction of the integrin with EGFR, thereby inhibiting growth and vasculogenic mimicry of triple-negative breast cancer cells ^117^.

Another example of integrin-targeting agents that may be clinically useful are the disintegrins, which are a family of small proteins found in snake venom that can target and block the adhesion functions of specific integrins (for a review on disintegrins and their potential use in cancer therapy, see 118). Indeed, therapeutic modulation of integrin signaling through the administration of disintegrins was recently shown to elicit anti-migratory and anti-angiogenic effects in both *in vitro* and *in vivo* cancer models ^118– 120^, indicating potential utility in the clinic.

In summary, while numerous studies have identified different integrin-targeting strategies, it is becoming increasingly clear that minding the TME will be critical for their effective implementation in the clinic. Moreover, it is highly likely that integrin-targeting agents will be most effective as part of a multi-combinatorial approach rather than as a monotherapy.

## Conclusions

The multifaceted nature of integrin biology is increasingly evident as more and more studies reveal the wide range of both distinct and overlapping roles that integrins play, individually and cooperatively, both in normal tissue remodeling and in pathological processes such as tumor progression and metastasis. When considering integrins as possible anti-cancer targets, the vast functionality of the integrin repertoire present on cancer cells, as well as on cells of the tumor stroma, provides considerable complexity even before considering the additional complexities of a dynamic TME. Indeed, the ever-evolving TME must be better understood, and we must take into account the repertoire of integrins that are expressed by distinct cells within the TME (as well as the availability of ligands for those integrins in the TME) while also keeping in mind that the function of a specific integrin may depend on cellular context. In addition, we must consider the abilities of some integrins to regulate paracrine communication between cells as well as the potential for combinatorial and compensatory functions of different integrins.

Despite the challenges raised by these complexities, as a group the integrins remain potentially powerful anti-cancer targets worthy of further investigation and exploitation in the clinic. Whether integrins will be useful as adjuvant targets or targets of monotherapies remains to be seen. In any case, the future of exploiting integrins as anti-cancer targets begins at the bench and will likely require a holistic approach that effectively considers the above-mentioned complexities of integrin biology within the context of an evolving TME. Achieving this goal will require not only the identification of the full spectrum of integrins expressed by both tumor cells and stromal cells within the TME but also the elucidation of how different integrins function coordinately and cooperatively within these cells. Such an approach should facilitate our ability to determine the most effective integrin combinations to target at the most appropriate stages of disease progression.

## Abbreviations

CAF, cancer-associated fibroblast; ECM, extracellular matrix; EGFR, epidermal growth factor receptor; FAK, focal adhesion kinase; IBD, inflammatory bowel disease; MAPK, mitogen-activated protein kinase; MMP, matrix metalloprotease; TAF, tumor-associated fibroblast; TAM, tumor-associated macrophage; TME, tumor microenvironment; uPAR, urokinase-type plasminogen activator receptor.
